# A Closed-Form Error Model of Straight Lines for Improved Data Association and Sensor Fusing

**DOI:** 10.3390/s18041236

**Published:** 2018-04-17

**Authors:** Volker Sommer

**Affiliations:** Department of Computer Science and Media, Beuth University of Applied Sciences, Luxemburger Str. 10, D-13353 Berlin, Germany; sommer@beuth-hochschule.de; Tel.: +49-30-4504-5154

**Keywords:** linear regression, covariance matrix, data association, sensor fusing, SLAM

## Abstract

Linear regression is a basic tool in mobile robotics, since it enables accurate estimation of straight lines from range-bearing scans or in digital images, which is a prerequisite for reliable data association and sensor fusing in the context of feature-based SLAM. This paper discusses, extends and compares existing algorithms for line fitting applicable also in the case of strong covariances between the coordinates at each single data point, which must not be neglected if range-bearing sensors are used. Besides, in particular, the determination of the covariance matrix is considered, which is required for stochastic modeling. The main contribution is a new error model of straight lines in closed form for calculating quickly and reliably the covariance matrix dependent on just a few comprehensible and easily-obtainable parameters. The model can be applied widely in any case when a line is fitted from a number of distinct points also without a priori knowledge of the specific measurement noise. By means of extensive simulations, the performance and robustness of the new model in comparison to existing approaches is shown.

## 1. Introduction

Contour points acquired by active sensors using sonar, radar or LiDAR [1], or extracted from image data [2,3], are a key source of information for mobile robots in order to detect obstacles or to localize themselves in known or unknown environments [4,5]. For this purpose, often, geometric features are extracted from raw data since in contrast to detailed contours, features are uniquely described just by a limited set of parameters, and their extraction works as additional filtering in order to improve reliability when dealing with sensor noise and masking [6]. However, the performance of feature-based localization or SLAM strongly depends on exact the determination of a feature vector *y* from measured raw data. Moreover, especially for data association, as well as for sensor fusing, not only the feature parameters are needed, but also a reliable estimation of their uncertainty is required. Generally, in the case of non-linear multi-sensor fusing, likelihood-based models can be applied (see [7]), which employ Bayesian filtering [8] or the calculation of entropies [9] to quantify uncertain information. Alternatively, especially for localization and map building, sensor fusing often is achieved with a Kalman filter (EKF). For this purpose, the covariance matrix *R* is required, which encapsulates the variances of the single elements in *y* and their dependencies.

This will be obvious if one looks at the standard algorithm for updating an estimated system state $\hat{x}$ typically by means of EKF; compare [10,11,12]: new measurements *y* are plausible if their deviations from expected measurements $\hat{y}=h\left(\hat{x}\right)$ dependent on the in general non-linear measurement model $h(\hat{x})$ are within a limited range. For exact calculation of this limit, usually the Mahalanobis metric is applied (see [11,13]), which considers the covariance matrix *S* of the innovation $\nu =y-\hat{y}$ with $S\;=\;R+H\cdot \hat{P}\cdot H^{T}$ dependent on *R*, the covariance matrix $\hat{P}$ of the system state and using $H=\nabla h(\hat{x})$. A new measurement *y* will be considered to relate to an already known feature vector $\hat{y}$ if its distance is below a given threshold $r_{th}$ with $\nu^{T}S^{-1}\nu <r_{th}^{2}$. Only in this case, the system state vector $\hat{x}$ can be updated by means of $\Delta \hat{x}=K\cdot \nu$ using the Kalman gain $K=\hat{P}\cdot H^{T}\;\cdot S$, again depending on the covariance matrix *R* of the measurements, while otherwise $\hat{x}$ and $\hat{P}$ are expanded by the new feature.

Thus, for reliable map building, errors in the step of data association should be strictly avoided by means of exact knowledge of the covariance matrix at each measurement, since otherwise, multiple versions of certain features would be included in the map, while other features erroneously are ignored.

Particularly in artificial environments, straight lines in a plane are frequently used as features, since these are defined by just two parameters and can be clearly and uniquely determined. In contrast to point features, lines in images are almost independent of illumination and perspective, and a number of measurements can be taken along their length to localize them accurately and to distinguish them from artifacts [14]. Moreover, already, a single line enables a robot to determine its orientation and perpendicular distance, which clearly improves localization accuracy. Thus, many tracking systems have been proposed based on line features, either using range-bearing scans [15,16] or applying visual servoing (see [17,18]), and also, recently, this approach has been successfully implemented [19,20,21]. However, due to missing knowledge of the covariance matrix, for data association, often, suboptimal solutions like the Euclidean distance in Hough space [15] or other heuristics are used [22].

Obviously, fitting data to a straight line is a well-known technique, addressed in a large number of papers [23,24,25] and textbooks [26,27,28]. In [29], a recent overview of algorithms in this field is outlined. As shown in [30,31], if linear regression is applied to data with uncertainties in the *x*- and *y*-direction, always both coordinates must be considered as random variables. In [32], Arras and Siegwart suggest an error model for range-bearing sensors including a covariance matrix, affected exclusively by noise in the radial direction. Pfister et al. introduce weights into the regression algorithm in order to determine the planar displacement of a robot from range-bearing scans [33]. In [34], a maximum likelihood approach is used to formulate a general strategy for estimating the best fitted line from a set of non-uniformly-weighted range measurements. Furthermore, merging of lines and approximating the covariance matrix from an iterative approach is considered. In [30], Krystek and Anton point out that the weighting factors of the single measurements depend on the orientation of a line, which therefore can only be determined numerically. This concept has been later extended to the general case with covariances existing between the coordinates of each data point [35].

Since linear regression is sensitive with respect to outliers, split-and-merge algorithms must be applied in advance, if a contour consists of several parts; see [36,37]. In cases of strong interference, straight lines can still be identified by Hough-transformation (compare [38,39,40]), or alternatively, RANSAC algorithms can be applied; see [41,42]. Although these algorithms work reliably, exact determination of line parameters and estimating their uncertainties still requires linear regression [43].

In spite of a variety of contributions in this field, a straightforward, yet accurate algorithm for determining the covariance matrix of lines reliably, quickly and independently of the a priori mostly unknown measurement noise is missing. In Section 4, such a model in closed-form is proposed depending on just a few clearly-interpretable and easily-obtainable parameters. Besides its low complexity and great clarity, the main advantage of the covariance matrix in closed form results from the fact that it can be calculated from the same data points as used for line fitting without the need to provide additional reliability information of the measurements, which in many cases is not available.

Beforehand, in the next two paragraphs, existing methods for the linear regression and calculation of the covariance matrix are reviewed with certain extensions focusing on the usage of range-bearing sensors, which cause strong covariances between the *x*- and *y*-coordinates. Based on these theoretical foundations, Section 5 exhibits detailed simulation results in order to compare the precision and robustness of the presented algorithms.

## 2. Determination of the Accurate Line Parameters

**Figure 1:**
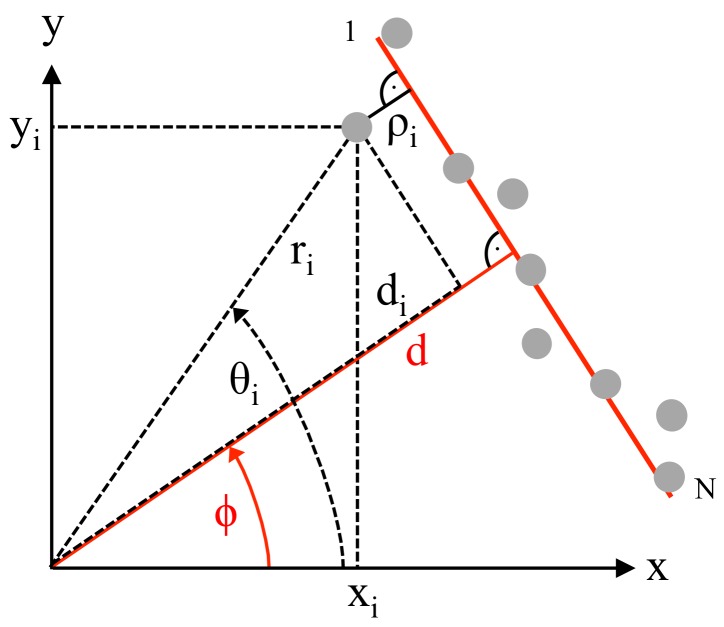
Parameters of measured raw data and a fitted straight line.

In 2D-space, each straight line is uniquely described by its perpendicular distance *d* from the origin and by the angle ϕ between the positive *x*-axis and this normal line; see Figure 1. In order to determine these two parameters, the mean squared error MSE considering the perpendicular distances of *N* measurement points from the fitted line needs to be minimized. For this purpose, each perpendicular distance $\rho_{i}$ of point *i* is calculated either from polar or with $x_{i}=r_{i}\cos\theta_{i}$ and $y_{i}=r_{i}\sin\theta_{i}$ alternatively in Cartesian coordinates as:

(1) $$\rho_{i}=d_{i}-d=r_{i}\cos\left(\theta_{i}-\phi \right)-d=x_{i}\cos\phi +y_{i}\sin\phi -d$$

Then, MSE is defined as follows dependent on ϕ and *d*:

(2) $$M\;S\;E\left(\phi ,d\right)=\sum_{i=1}^{N}{\left(s_{i}\rho_{i}\right)}^{2}$$

In (2), optional scaling values $s_{i}$ are included in order to consider the individual reliability of each measurement point. By calculating the derivatives of (2) with respect to ϕ and *d* and setting both to zero, the optimum values of these parameters can be analytically derived assuming all $s_{i}$ to be constant, i.e., independent of ϕ and *d*. The solution has been published elsewhere (compare [32]), and in the Appendix of this paper, a straightforward derivation is sketched, yielding for ϕ and *d*:

(3) $$\phi =\frac{1}{2}\cdot atan2\left(-2\sigma_{xy},\sigma_{y}^{2}-\sigma_{x}^{2}\right)$$

(4) $$d=\bar{x}\cos\phi +\bar{y}\sin\phi$$

The function atan2() means the four quadrant arc tangent, which calculates ϕ always in the correct range. If *d* becomes negative, its modulus must be taken, and the corresponding ϕ has to be altered by plus or minus π. In these equations, $\bar{x}$ and $\bar{y}$ denote the mean values of all *N* measurements $x_{i}$ and $y_{i}$, while $\sigma_{x}^{2}$, $\sigma_{y}^{2}$ and $\sigma_{xy}$ denote the variances and the covariance:

(5) $$\sigma_{x}^{2}=\frac{1}{N}\;\sum_{i=1}^{N}w_{i}{\left(x_{i}-\bar{x}\right)}^{2}$$

(6) $$\sigma_{y}^{2}=\frac{1}{N}\;\sum_{i=1}^{N}w_{i}{\left(y_{i}-\bar{y}\right)}^{2}$$

(7) $$\sigma_{xy}=\frac{1}{N}\;\sum_{i=1}^{N}w_{i}\left(x_{i}-\bar{x}\right)\left(y_{i}-\bar{y}\right)$$

(8) $$\bar{x}=\frac{1}{N}\;\sum_{i=1}^{N}w_{i}x_{i}$$

(9) $$\bar{y}=\frac{1}{N}\;\sum_{i=1}^{N}w_{i}y_{i}$$

In (5)–(9), normalized weighting factors $w_{i}$ are used with $\frac{1}{N}\sum_{i=1}^{N}w_{i}=1$ and $0\leq w_{i}\leq 1$, calculated dependent on the chosen scaling values $s_{i}$:

(10) $$w_{i}=\frac{s_{i}^{2}}{\frac{1}{N}\sum_{i=1}^{N}s_{i}^{2}}$$

**Figure 2:**
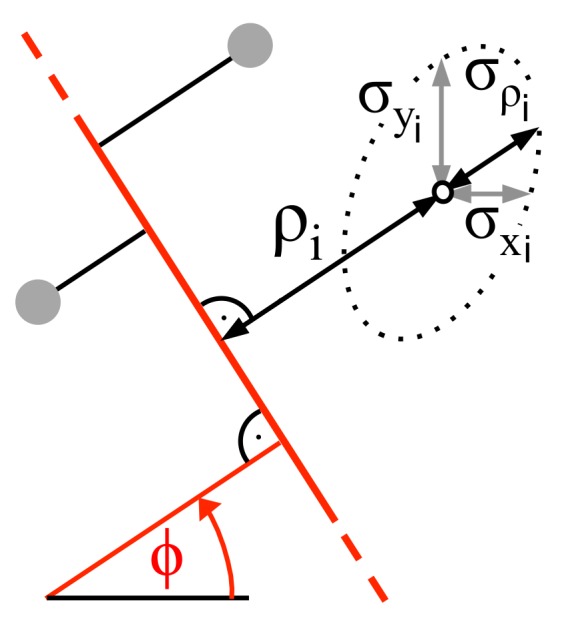
Optimum setting of weighting parameter for each data point.

As pointed out in [35], for accurate line matching, the scaling values $s_{i}$ must not be assumed to be constant since in general, they depend on ϕ. This can be understood from Figure 2, which shows for one measurement point *i* the error ellipse spanned by the standard deviations $\sigma_{x,i}$ and $\sigma_{y,i}$, while the rotation of the ellipse is caused by the covariance $\sigma_{xy,i}$.

Apparently, as a measure of confidence, only the deviation $\sigma_{\rho ,i}$ perpendicular to the line is relevant, while the variance of any data point parallel to the fitted line does not influence its reliability. Thus, the angle ϕ given in (3) will only be exact, if the error ellipse equals a circle, which means that all measurements exhibit the same standard deviations in the *x*- as in the *y*-direction, and no covariance exists. Generally, in order to determine optimum line parameters with arbitrary variances and covariance of each measurement *i*, in Equation (2) the inverse of $\sigma_{\rho ,i}$ dependent on ϕ has to be used as scaling factor $s_{i}$, yielding:

(11) $$M\;S\;E\left(\phi \right)=\sum_{i=1}^{N}\frac{\rho_{i}^{2}\left(\phi \right)}{\sigma_{\rho ,i}^{2}\left(\phi \right)}$$

In this formula, which can only be solved numerically, the variance $\sigma_{\rho ,i}^{2}$ needs to be calculated dependent on the covariance matrix of each measurement point *i*. In the case of line fitting from range-bearing scans, the covariance matrix ${\underline{R}}_{r\theta ,i}$ can be modeled as a diagonal matrix since both parameters $r_{i}$ and $\theta_{i}$ are measured independently, and thus, their covariance $\sigma_{r\theta ,i}$ equals zero:

(12) $${\underline{R}}_{r\theta ,i}=\left(\begin{array}{cc} \sigma_{r,i}^{2} & 0\\ 0 & \sigma_{\theta ,i}^{2} \end{array}\right)$$

Typically, this matrix may also be considered as constant, thus independent of index *i*, assuming that all measured radii and angles are affected by the same noise, i.e., ${\underline{R}}_{r\theta ,i}\approx {\underline{R}}_{r\theta }$.

With known variances $\sigma_{r,i}^{2}$ and $\sigma_{\theta ,i}^{2}$ and for a certain ϕ, now $\sigma_{\rho ,i}^{2}$ is determined by evaluating the relation between $\rho_{i}$ and the distances $d_{i}$ of each data point with 1≤i≤N. According to (1) and with the distance *d* written as the mean of all $d_{i}$, it follows:

(13) $$\rho_{i}=d_{i}-\frac{1}{N}\;\sum_{j=1}^{N}d_{j}=\left(\frac{N-1}{N}\right)d_{i}-\frac{1}{N}\;\sum_{\begin{array}{c} j=1\\ \left(j\neq i\right) \end{array}}^{N}d_{j}$$

Since noise-induced variations of all distances $d_{i}$ are uncorrelated with each other, now the variance $\sigma_{\rho ,i}^{2}$ is calculated by means of summing over all variances $\sigma_{d,i}^{2}$:

(14) $$\sigma_{\rho ,i}^{2}={\left(\frac{N-1}{N}\right)}^{2}\sigma_{d,i}^{2}+\frac{1}{N^{2}}\;\sum_{\begin{array}{c} j=1\\ \left(j\neq i\right) \end{array}}^{N}\sigma_{d,j}^{2}$$

In order to derive $\sigma_{d,i}^{2}$, changes of $d_{i}$ with respect to small deviations of $r_{i}$ and $\theta_{i}$ from their expected values ${\bar{r}}_{i}$ and ${\bar{\theta }}_{i}$ are considered with $d_{i}={\bar{d}}_{i}+\Delta d_{i}$, $r_{i}={\bar{r}}_{i}+\Delta r_{i}$ and with $\theta_{i}={\bar{\theta }}_{i}+\Delta \theta_{i}$:

(15) $$\Delta d_{i}=\Delta d_{i}^{\;r}+\Delta d_{i}^{\;\theta }$$

The terms on the right side of (15) can be determined independently of each other, since $\Delta r_{i}$ and $\Delta \theta_{i}$ are assumed to be uncorrelated. With $d_{i}=r_{i}\cdot \cos\left(\theta_{i}-\phi \right)$, it follows:

(16) $$\Delta d_{i}^{\;r}=\Delta r_{i}\cdot \cos\left({\bar{\theta }}_{i}-\phi \right)$$

and:

(17) $$\Delta d_{i}^{\;\theta }={\bar{r}}_{i}\left[\cos\left({\bar{\theta }}_{i}-\phi +\Delta \theta_{i}\right)-\cos\left({\bar{\theta }}_{i}-\phi \right)\right]\approx -{\bar{r}}_{i}\;\left[\frac{\Delta \theta_{i}^{2}}{2}\cos\left({\bar{\theta }}_{i}-\phi \right)+\Delta \theta_{i}\sin\left({\bar{\theta }}_{i}-\phi \right)\right]$$

In the last line, the addition theorem was applied for cos$\left({\bar{\theta }}_{i}-\phi +\Delta \theta_{i}\right)$, and for small variations, the approximations cos$\left(\Delta \theta_{i}\right)\;\approx \;1-\frac{\Delta \theta_{i}^{2}}{2}$ and sin$\left(\Delta \theta_{i}\right)\;\approx \;\Delta \theta_{i}$ are valid.

The random variables $\Delta r_{i}$ and $\Delta \theta_{i}$ are assumed to be normally distributed with variances $\sigma_{r,i}^{2}$ and $\sigma_{\theta ,i}^{2}$. Thus, the random variable $\Delta \theta_{i}^{2}$ exhibits a $\chi^{2}$-distribution with variance $2{\left(\sigma_{\theta ,i}^{2}\right)}^{2}$ (see [44]), and the variance of $d_{i}$ is calculated from (15)–(17) as the weighted sum with ${\bar{r}}_{i}$ and ${\bar{\theta }}_{i}$ approximately replaced by $r_{i}$ and $\theta_{i}$, respectively:

(18) $$\sigma_{d,i}^{2}=\left(\sigma_{r,i}^{2}+\frac{{\left(\sigma_{\theta ,i}^{2}\right)}^{2}}{2}\right)\cos^{2}\left(\theta_{i}-\phi \right)+\sigma_{\theta ,i}^{2}\sin^{2}\left(\theta_{i}-\phi \right)$$

When applying this algorithm, a one-dimensional minimum search of MSE according to (11) needs to be executed, yielding the optimum ϕ of the straight line. For this purpose, $\sigma_{\rho ,i}^{2}$ is inserted from (14) considering (18) and $\rho_{i}$ is determined according to (1) by calculating *d* from (4) and (8)–(10) with $s_{i}=1/\sigma_{\rho ,i}$.

Obviously, numerical line fitting can also be accomplished if measurements are available in Cartesian coordinates $x_{i}$ and $y_{i}$. In this case, the covariance matrix ${\underline{R}}_{xy,i}$ of each measurement point must be known, defined as:

(19) $${\underline{R}}_{xy,i}=\left(\begin{array}{cc} \sigma_{x,i}^{2} & \sigma_{xy,i}\\ \sigma_{xy,i} & \sigma_{y,i}^{2} \end{array}\right)$$

Furthermore, the partial derivatives of $d_{i}$ according to (1) with respect to $x_{i}$ and $y_{i}$ need to be calculated:

(20) $${\underline{J}}_{d,i}=\left(\begin{array}{cc} \frac{\partial d_{i}}{\partial x_{i}} & \frac{\partial d_{i}}{\partial y_{i}} \end{array}\right)=\left(\begin{array}{cc} \cos\phi & \sin\phi \end{array}\right)$$

Then, $\sigma_{d,i}^{2}$ follows dependent on ${\underline{R}}_{xy,i}$ and ${\underline{J}}_{d,i}$:

(21) $$\sigma_{d,i}^{2}={\underline{J}}_{d,i}\cdot {\underline{R}}_{xy,i}\cdot {\left({\underline{J}}_{d,i}\right)}^{T}=\sigma_{x,i}^{2}\cos^{2}\phi +\sigma_{xy,i}\sin\phi \cos\phi +\sigma_{y,i}^{2}\sin^{2}\phi$$

If raw data stem from a range-bearing scan, ${\underline{R}}_{xy,i}$ can be calculated from ${\underline{R}}_{r\theta ,i}$ by exploiting the known dependencies between the polar and Cartesian plane. For this purpose, the Jacobian matrix ${\underline{J}}_{xy,i}$ is determined:

(22) $${\underline{J}}_{xy,i}=\left(\begin{array}{cc} \frac{\partial x_{i}}{\partial r_{i}} & \frac{\partial x_{i}}{\partial \theta_{i}}\\ \frac{\partial y_{i}}{\partial r_{i}} & \frac{\partial y_{i}}{\partial \theta_{i}} \end{array}\right)=\left(\begin{array}{cc} \cos\theta_{i} & -r_{i}\sin\theta_{i}\\ \sin\theta_{i} & r_{i}\cos\theta_{i} \end{array}\right)$$

Then, the covariance matrix ${\underline{R}}_{xy,i}$ will depend on ${\underline{R}}_{r\theta ,i}$, if small deviations from the mean value of the random variables $r_{i}$ and $\theta_{i}$ and a linear model are assumed:

(23) $${\underline{R}}_{xy,i}={\underline{J}}_{xy,i}\cdot {\underline{R}}_{r\theta ,i}\cdot {\left({\underline{J}}_{xy,i}\right)}^{T}$$

According to (23), generally a strong covariance $\sigma_{xy,i}$ in ${\underline{R}}_{xy,i}$ must be considered, if measurements are taken by range-bearing sensors.

By means of applying (21)–(23) instead of (18) for searching the minimum of MSE dependent on ϕ, the second order effect regarding $\Delta \theta_{i}$ is neglected. This yields almost the same formula as given in [35], though the derivation differs, and in [35], additionally, the variance of *d* is ignored assuming $\sigma_{\rho ,i}^{2}=\sigma_{d,i}^{2}$, which according to (14) is only asymptotically correct for large *N*.

Finally, it should be noted that the numerical determination of ϕ according to (11) means clearly more complexity compared to the straightforward solution according to Equation (3). Later, in Section 5, it will be analyzed under which conditions this additional computational effort actually is required.

## 3. Analytic Error Models of Straight Lines

In the literature, several methods are described to estimate errors of ϕ and *d* and their mutual dependency. Thus, the covariance matrix $R_{d\phi }$ must be known, defined as:

(24) $${\underline{R}}_{d\phi }=\left(\begin{array}{cc} \sigma_{d}^{2} & \sigma_{d\phi }\\ \sigma_{d\phi } & \sigma_{\phi }^{2} \end{array}\right)$$

For this purpose, a general method in nonlinear parameter estimation is the calculation of the inverse Hessian matrix at the minimum of MSE. Details can be found in [30,35], while in [45], it is shown that this procedure may exhibit numerical instability. In Section 5, results using this method are compared with other approaches.

Alternatively, in [32,46], an analytic error model is proposed based on fault analysis of the line parameters. In this approach, the effect of variations of each single measurement point defined by ${\underline{R}}_{xy,i}$ with respect to the covariance matrix of the line parameters ${\underline{R}}_{d\phi }$ is considered, based on (3) and (4). Thereto, the Jacobian matrix ${\underline{J}}_{d\phi ,i}$ with respect to $x_{i}$ and $y_{i}$ is determined, defined as:

(25) $${\underline{J}}_{d\phi ,i}=\left(\begin{array}{cc} \frac{\partial d}{\partial x_{i}} & \frac{\partial d}{\partial y_{i}}\\ \frac{\partial \phi }{\partial x_{i}} & \frac{\partial \phi }{\partial y_{i}} \end{array}\right)$$

With this matrix, the contribution of a single data point *i* to the covariance matrix between *d* and ϕ can be written as:

(26) $${\underline{R}}_{d\phi ,i}={\underline{J}}_{d\phi ,i}\cdot {\underline{R}}_{xy,i}\cdot {\underline{J}}_{d\phi ,i}^{T}$$

For determining the partial derivatives of *d* in (25), Equation (4) is differentiated after expanding it by (8) and (9), yielding:

(27) $$\frac{\partial d}{\partial x_{i}}=w_{i}\frac{\cos\phi }{N}+\left(\bar{y}\cos\phi -\bar{x}\sin\phi \right)\frac{\partial \phi }{\partial x_{i}}$$

(28) $$\frac{\partial d}{\partial y_{i}}=w_{i}\frac{\sin\phi }{N}+\left(\bar{y}\cos\phi -\bar{x}\sin\phi \right)\frac{\partial \phi }{\partial y_{i}}$$

Differentiating ϕ according to (3) with respect to $x_{i}$ gives the following expression with $u=-2\sigma_{xy}$ and $v=\sigma_{y}^{2}-\sigma_{x}^{2}$:

(29) $$\frac{\partial \phi }{\partial x_{i}}=\frac{1}{2(u^{2}+v^{2})}\left(\frac{\partial u}{\partial x_{i}}v-\frac{\partial v}{\partial x_{i}}u\right)$$

The partial derivation of *u* in (29) is calculated after expanding it with (7) and (8) as:

(30) $$\frac{\partial u}{\partial x_{i}}=-\frac{2}{N}\cdot \frac{\partial }{\partial x_{i}}\left(\sum_{i=1}^{N}w_{i}x_{i}y_{i}-\bar{y}\sum_{i=1}^{N}w_{i}x_{i}\right)=-\frac{2w_{i}}{N}\left(y_{i}-\bar{y}\right)$$

while partial derivation of *v* with (5), (6) and (8) yields:

(31) $$\frac{\partial v}{\partial x_{i}}=-\frac{1}{N}\cdot \frac{\partial }{\partial x_{i}}\left(\sum_{i=1}^{N}w_{i}x_{i}^{2}-\frac{1}{N}{\left(\sum_{i=1}^{N}w_{i}x_{i}\;\right)}^{2}\right)=-\frac{2w_{i}}{N}\left(x_{i}-\bar{x}\right)$$

Finally, after substituting all terms with *u* and *v* in (29), it follows:

(32) $$\frac{\partial \phi }{\partial x_{i}}=w_{i}\frac{\left(\sigma_{x}^{2}-\sigma_{y}^{2}\right)\left(y_{i}-\bar{y}\right)-2\sigma_{xy}\left(x_{i}-\bar{x}\right)}{N\left({\left(\sigma_{x}^{2}-\sigma_{y}^{2}\right)}^{2}+4\sigma_{xy}^{2}\right)}$$

Correspondingly, for the partial derivative of ϕ with respect to $y_{i}$, the following result is obtained:

(33) $$\frac{\partial \phi }{\partial y_{i}}=w_{i}\frac{\left(\sigma_{x}^{2}-\sigma_{y}^{2}\right)\left(x_{i}-\bar{x}\right)+2\sigma_{xy}\left(y_{i}-\bar{y}\right)}{N\left({\left(\sigma_{x}^{2}-\sigma_{y}^{2}\right)}^{2}+4\sigma_{xy}^{2}\right)}$$

Now, after inserting (27), (28), (32) and (33) into (25), the covariance matrix of *d* and ϕ (24) is calculated by summing over all *N* data points since the noise contributions of the single measurements can be assumed to be stochastically independent of each other:

(34) $${\underline{R}}_{d\phi }=\sum_{i=1}^{N}{\underline{R}}_{d\phi ,i}=\sum_{i=1}^{N}{\underline{J}}_{d\phi ,i}\cdot {\underline{R}}_{xy,i}\cdot {\underline{J}}_{d\phi ,i}^{T}$$

Equation (34) enables an exact calculation of the variances $\sigma_{d}^{2}$, $\sigma_{\phi }^{2}$ and of the covariance $\sigma_{d\phi }$ as long as the deviations of the measurements stay within the range of a linear approach, and as long as Equations (3) and (4) are valid. In contrast to the method proposed in [35], no second derivatives and no inversion of the Hessian matrix are needed, and thus, more stable results can be expected.

However, both algorithms need some computational effort especially for a large number of measurement points. Moreover, they do not allow one to understand the effect of changing parameters on $R_{d\phi }$, and these models can only be applied, if for each data point, the covariance matrix ${\underline{R}}_{xy,i}$ is available. Unfortunately, for lines extracted from images, this information is unknown, and also, in the case of using range-bearing sensors, only a worst case estimate of $\sigma_{r}$ is given in the data sheet, while $\sigma_{\theta }$ is ignored.

## 4. Closed-Form Error Model of a Straight Line

In this section, a simplified error model in closed form is deduced, which enables a fast, clear and yet, for most applications, sufficiently accurate calculation of the covariance matrix ${\underline{R}}_{d\phi }$ in any case when line parameters *d* and ϕ have been determined from a number of discrete data points.

Thereto, first, the expected values of the line parameters *d* and ϕ, denoted as $\bar{d}$ and $\bar{\phi }$, are assumed to be known according to the methods proposed in Section 2 with $\bar{d}\approx d$ and $\bar{\phi }\approx \phi$. Besides, for modeling the small deviation of *d* and ϕ, the random variables Δd and Δϕ are introduced. Thus, with $d=\bar{d}+\Delta d$ and $\phi =\bar{\phi }+\Delta \phi$, it follows for the variances and the covariance:

(35) $$\sigma_{d}^{2}=\sigma_{\Delta d}^{2}\;\sigma_{\phi }^{2}=\sigma_{\Delta \phi }^{2}\;\sigma_{d\phi }=\sigma_{\Delta d\Delta \phi }$$

**Figure 3:**
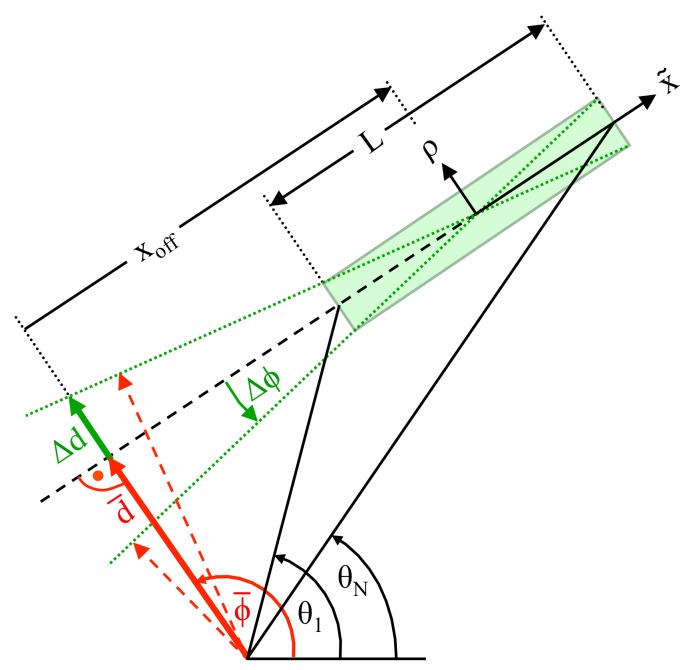
Dependency between Δd, Δϕ and geometric parameters.

Next, Δd and Δϕ shall be determined dependent on a random variation of any of the *N* measured data points. For this purpose, Figure 3 is considered, which shows the expected line parameters and the random variables Δd and Δϕ.

**Figure 4:**
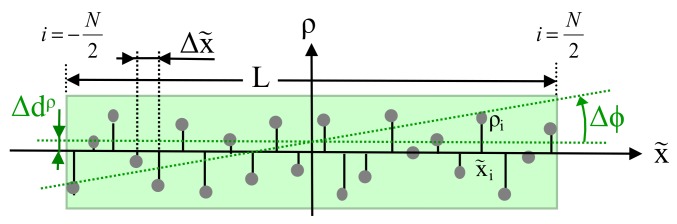
Details of Figure 3 with the deviation of data points along the axis $\tilde{x}$.

In order to derive expressions for Δd and Δϕ depending on the random variables $\rho_{i}$, Figure 4 shows an enlargement of the rectangular box depicted in Figure 3 along the direction of the line $\tilde{x}$.

First, the effect of variations of any $\rho_{i}$ on Δϕ is considered. Since Δϕ is very small, this angle may be replaced by its tangent, which defines the slope Δm of the line with respect to the direction $\tilde{x}$. Here, only $\rho_{i}$ is considered as random variable, but not ${\tilde{x}}_{i}$. Thus, the standard formula for the slope of a regression line can be applied (see, e.g., [26] Chapter 2), which will minimize the mean squared distance in the direction of ρ, if all ${\tilde{x}}_{i}$ are assumed to be exactly known:

(36) $$\Delta \phi \approx \tan\left(\Delta \phi \right)=\Delta m=\frac{\sigma_{\rho \tilde{x}}}{\sigma_{\tilde{x}}^{2}}=\frac{\sum_{i}\rho_{i}\cdot {\tilde{x}}_{i}}{\sum_{i}{\tilde{x}}_{i}^{2}}$$

Now, in order to calculate the variance of Δϕ, a linear relation between Δϕ and each $\rho_{i}$ is required, which is provided by the first derivation of (36) with respect to $\rho_{i}$:

(37) $$\frac{\partial \Delta \phi }{\partial \rho_{i}}=\frac{{\tilde{x}}_{i}}{\sum_{i}{\tilde{x}}_{i}^{2}}$$

Then, the variance of Δϕ dependent on the variance of $\rho_{i}$ can be specified. From (37), it follows:

(38) $$\sigma_{\Delta \phi ,i}^{2}=\sigma_{\rho ,i}^{2}\cdot {\left(\frac{\partial \Delta \phi }{\partial \rho_{i}}\right)}^{2}=\sigma_{\rho ,i}^{2}\cdot \frac{{\tilde{x}}_{i}^{2}}{{\left(\sum_{i}{\tilde{x}}_{i}^{2}\right)}^{2}}$$

If $\sigma_{\rho ,i}^{2}$ is assumed to be approximately independent of *i*, it may be replaced by $\sigma_{\rho }^{2}$ and can be estimated from (2) with $\rho_{i}$ taken from (1) and setting all $s_{i}$ to 1/N:

(39) $$\sigma_{\rho ,i}^{2}\approx \sigma_{\rho }^{2}=\frac{1}{N}\sum_{i=1}^{N}\rho_{i}{\left(\phi ,d\right)}^{2}$$

It should be noted that for a bias-free estimation of $\sigma_{\rho }^{2}$ with (39), the exact line parameters ϕ and *d* must be used in (1), which obviously are not available. If instead, estimated line parameters according to Section 2 are taken, e.g., by applying (3) and (4), calculated from the same data as used in (39), an underestimation of $\sigma_{\rho }^{2}$ especially for small *N* can be expected, since ϕ and *d* are determined by minimizing the variance of ρ of these *N* data points. This is referred to later.

Next, from (38), the variance of Δϕ results as the sum over all *N* data points, since all $\rho_{i}$ are independent of each other:

(40) $$\sigma_{\Delta \phi }^{2}=\sum_{i}\sigma_{\Delta \phi ,i}^{2}\approx \sigma_{\rho }^{2}\cdot \frac{\sum_{i}{\tilde{x}}_{i}^{2}}{{\left(\sum_{i}{\tilde{x}}_{i}^{2}\right)}^{2}}=\sigma_{\rho }^{2}\cdot \frac{1}{\sum_{i}{\tilde{x}}_{i}^{2}}$$

Equations (40) with (35) and (39) enables an exact calculation of $\sigma_{\phi }^{2}$ dependent on the *N* data points of the line.

However, from (40), a straightforward expression can be derived, which is sufficiently accurate in most cases and enables a clear understanding of the influencing parameters on $\sigma_{\phi }^{2}$; compare Section 5. For this purpose, according to Figure 3, the length *L* of a line segment is determined from the perpendicular distance *d* and from the angles $\theta_{1}$ and $\theta_{N}$ of the first and *N*-th data point, respectively:

(41) $$L=d\cdot \left|\tan\left(\phi -\theta_{N}\right)-\tan\left(\phi -\theta_{1}\right)\right|$$

Furthermore, a constant spacing $\Delta \tilde{x}$ between adjacent data points is assumed:

(42) $$\Delta \tilde{x}\approx \frac{L}{N-1}.$$

Applying this approximation, the sum over all squared ${\tilde{x}}_{i}$ can be rewritten, yielding for even *N* as depicted in Figure 4:

(43) $$\sum_{i}{\tilde{x}}_{i}^{2}\approx 2\cdot \sum_{i=1}^{N/2}{\left[\frac{\Delta \tilde{x}}{2}\left(2i-1\right)\right]}^{2}=\Delta {\tilde{x}}^{2}\cdot \sum_{i=1}^{N/2}\frac{{\left(2i-1\right)}^{2}}{2}$$

The last sum can be transformed into closed form as:

(44) $$\sum_{i=1}^{N/2}\frac{{\left(2i-1\right)}^{2}}{2}=\frac{\frac{N}{2}\left(4{\left(\frac{N}{2}\right)}^{2}\;\;-1\right)}{6}=\frac{N(N^{2}-1)}{12}$$

With *N* odd, the sum must be taken twice from 1–$\frac{N-1}{2}$, since in this case, the central measurement point has no effect on $\sigma_{\Delta \phi ,i}^{2}$, yielding:

(45) $$\sum_{i}{\tilde{x}}_{i}^{2}\approx 2\cdot \;\;\;\sum_{i=1}^{\frac{\left(N-1\right)}{2}}{\left[\Delta \tilde{x}\cdot i\right]}^{2}=\Delta {\tilde{x}}^{2}\cdot \;\;\;\sum_{i=1}^{\frac{\left(N-1\right)}{2}}2\cdot i^{2}$$

Again, the last sum can be written in closed form, which gives the same result as in (44):

(46) $$\sum_{i=1}^{\frac{\left(N-1\right)}{2}}2\cdot i^{2}=\frac{\frac{N-1}{2}\left(\frac{N-1}{2}+1\right)\left(2\cdot \frac{N-1}{2}+1\right)}{3}=\frac{N(N^{2}-1)}{12}$$

Finally, by substituting (43) with (44) or (45) with (46) into (40) and regarding (35), as well as (42), a simple analytic formula for calculating the variance of ϕ is obtained, just depending on *L*, *N* and the variance of ρ:

(47) $$\sigma_{\phi }^{2}\approx \sigma_{\rho }^{2}\cdot \frac{12}{L^{2}\cdot N}\cdot \frac{N-1}{N+1}\overset{N\gg 1}{\approx }\sigma_{\rho }^{2}\cdot \frac{12}{L^{2}\cdot N}$$

The last simplification in (47) overestimates $\sigma_{\phi }^{2}$ a little bit for small *N*. Interestingly, this error compensates quite well for a certain underestimation of $\sigma_{\rho }^{2}$ according to (39), assuming that the line parameters ϕ and *d* are determined from the same data as $\sigma_{\rho }^{2}$; see Section 5.

Next, in order to deduce the variance $\sigma_{d}^{2}$, again, Figure 3 is considered. Apparently, the first part of the random variable Δd is strongly correlated with Δϕ since any mismatch in ϕ is transformed into a deviation Δd by means of the geometric offset $x_{off}$ with:

(48) $$\Delta d^{\;\phi }=-x_{off}\cdot \Delta \phi$$

Actually, with a positive value for $x_{off}$, as depicted in Figure 3 the correlation between Δd and Δϕ becomes negative, since positive values of Δϕ correspond to negative values of Δd. According to Figure 3, $x_{off}$ is determined from ϕ and *d*, as well as from $\theta_{1}$ and $\theta_{N}$:

(49) $$x_{off}=\frac{d}{2}\cdot \left[\tan\left(\phi -\theta_{N}\right)+\tan\left(\phi -\theta_{1}\right)\right]$$

Alternatively, $x_{off}$ can be taken as the mean value from all *N* data points of the line segment:

(50) $$x_{off}=\frac{d}{N}\cdot \sum_{i=1}^{N}\tan\left(\phi -\theta_{i}\right)$$

Nevertheless, it should be noted that Δd is not completely correlated with Δϕ, since also in the case $x_{off}=0$, the error Δd will not be zero.

Indeed, as a second effect, each single $\rho_{i}$ has a direct linear impact on the variable Δd. For this purpose, in Figure 4, the random variable $\Delta d^{\;\rho }$ is depicted, which describes a parallel shift of the regression line due to variation in $\rho_{i}$, calculated as the mean value over all $\rho_{i}$:

(51) $$\Delta d^{\;\rho }=\frac{1}{N}\cdot \sum_{i}\rho_{i}$$

Combining both effects, variations in *d* can be described as the sum of two uncorrelated terms, $\Delta d^{\phi }$ and $\Delta d^{\rho }$:

(52) $$\Delta d=\Delta d^{\;\phi }+\Delta d^{\;\rho }=-x_{off}\cdot \Delta \phi +\frac{1}{N}\cdot \sum_{i}\rho_{i}$$

This missing correlation between Δϕ and the sum over all $\rho_{i}$ is also intuitively accessible: if the latter takes a positive number, it will not be possible to deduce the sign or the modulus of Δϕ. From (52) and with $E(\Delta d^{\;\phi }\;\cdot \;\Delta d^{\;\rho })=0$, $E(\Delta d^{\;\phi })=0$ and $E(\Delta d^{\;\rho })=0$, the variance $\sigma_{d}^{2}$ can be calculated as:

(53) $$\sigma_{d}^{2}=E\left({\left[\Delta d\right]}^{2}\right)=E\left({\left[\Delta d^{\;\phi }\right]}^{2}\right)+E\left({\left[\Delta d^{\;\rho }\right]}^{2}\right)=x_{off}^{2}\cdot E\left({\left[\Delta \phi \right]}^{2}\right)+\frac{1}{N^{2}}\cdot E\left({\left[\sum_{i}\rho_{i}\right]}^{2}\right)$$

(54) $$\approx x_{off}^{2}\cdot \sigma_{\phi }^{2}+\frac{1}{N}\cdot \sigma_{\rho }^{2}$$

In the last step from (53) to (54), again, the independence of the single measurements from each other is used; thus, the variance of the sum of the *N* data points approximates *N*-times the variance $\sigma_{\rho }^{2}$.

Finally, the covariance between ϕ and *d* needs to be determined. Based on the definition, it follows with $\sigma_{d\phi }=\sigma_{\Delta d\Delta \phi }$:

(55) $$\sigma_{d\phi }=E\left(\Delta d\cdot \Delta \phi \right)=E\left(\Delta d^{\;\phi }\cdot \Delta \phi \right)+E\left(\Delta d^{\;\rho }\cdot \Delta \phi \right)=-x_{off}\cdot E\left({\left[\Delta \phi \right]}^{2}\right)=-x_{off}\cdot \sigma_{\phi }^{2}$$

By means of (47), (54) and (55), now, the complete error model in closed form is known, represented by the covariance matrix ${\underline{R}}_{d\phi }$ given as:

(56) $${\underline{R}}_{d\phi }\approx \sigma_{\rho }^{2}\cdot \left(\begin{array}{cc} \frac{12\cdot x_{off}^{2}}{L^{2}\cdot N}+\frac{1}{N} & \frac{-12\cdot x_{off}}{L^{2}\cdot N}\\ \frac{-12\cdot x_{off}}{L^{2}\cdot N} & \frac{12}{L^{2}\cdot N} \end{array}\right)$$

Applying this error model is easy since no knowledge of the variances and covariance for each single measurement is needed, which in practice is difficult to acquire. Instead, just the number *N* of preferably equally-spaced points used for line fitting, the variance $\sigma_{\rho }^{2}$ according to (39), the length *L* of the line segment calculated with (41) and its offset $x_{off}$ according to (49) or (50) must be inserted.

## 5. Simulation Results

The scope of this section is to compare the presented algorithms for linear regression and error modeling based on statistical evaluation of the results. Segmentation of raw data is not considered; if necessary, this must be performed beforehand by means of well-known methods like Hough transformation or RANSAC; compare Section 1. Thus, for studying the performance reliably and repeatably, a large number of computer simulations was performed, applying a systematic variation of parameters within a wide range, which would not be feasible if real measurements are used.

**Figure 5:**
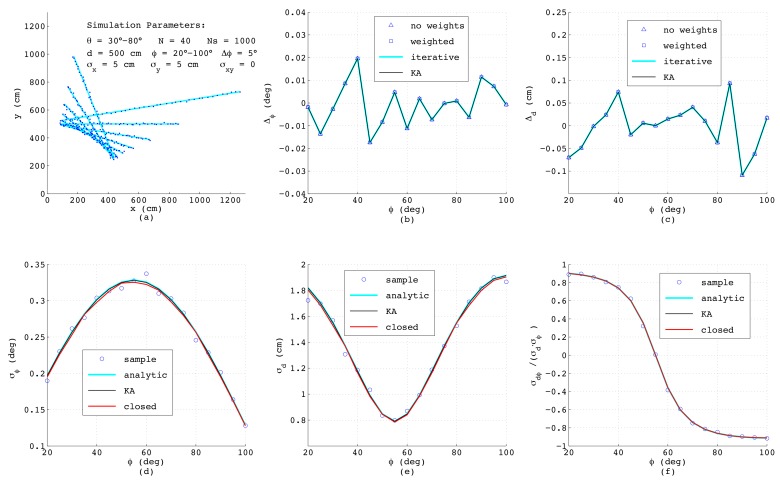
Simulation results for equidistant measurement points superimposing normally-distributed and uncorrelated noise in the *x*- and *y*-direction.

For this purpose, straight lines with a certain perpendicular distance *d* from the origin and within a varying range of normal angles ϕ have been specified. Each of these lines is numerically described by a number of *N* points either given in Cartesian ($x_{i},y_{i}$) or in polar ($r_{i}$$\theta_{i}$) coordinates. In order to simulate the outcome of a real range-bearing sensor as much as possible, the angular coordinate was varied between $\theta_{1}$ and $\theta_{N}$. To each measurement, a certain amount of normally-distributed noise with $\sigma_{x}$, $\sigma_{y}$ and $\sigma_{xy}$ or alternatively with $\sigma_{r}$ and $\sigma_{\theta }$ was added. Further, for each ϕ, a number of $N_{s}=1000$ sets of samples was generated, in order to allow statistical evaluation of the results. The first simulation was performed with N=40 equally-spaced points affected each by uncorrelated noise in the *x*- and *y*-direction with standard deviations $\sigma_{x}\;=\;\sigma_{y}\;=\;5\;$ cm. This is a typical situation when a line is calculated from binary pixels, and in Figure 5a, a bundle of the simulated line segments is shown. The deviations $\Delta_{\phi }$ and $\Delta_{d}$ taken as the mean value over all $N_{s}$ samples of the estimated ϕ and *d* from their true values, respectively, are depicted in Figure 5b,c, comparing four algorithms as presented in Section 2: The triangles mark the outcome of Equations (3) and (4) with all weights set to one, whereas the squares are calculated according to the same analytic formulas, but using individual weighting factors applying (10) with $s_{i}=1/\sigma_{\rho ,i}$. The perpendicular deviations $\sigma_{\rho ,i}$ are determined according to (14) and (21) with ϕ taken from (3) without weights. Obviously, in this example, all triangles coincide with the squares since each measurement *i* is affected by the same noise and thus for any ϕ, all weighting factors are always identical. The blue lines in Figure 5b,c show the results when applying the iterative method according to (11) with the minimum of MSE found numerically. For this purpose, $\sigma_{\rho ,i}^{2}$ is inserted from (14) considering (21) and $\rho_{i}$ is taken from (1) and *d* is calculated from (4), (8)–(10) with $s_{i}=1/\sigma_{\rho ,i}$. The black lines (KA) depict the deviations of *d* and ϕ obtained according to Krystek and Anton in [35]. Both numerical algorithm yield the same results, which is not surprising, since the variances $\sigma_{\rho ,i}^{2}$ used as weighting factors are all identical. Further, here, the analytical algorithms provide exactly the same performance as the numerical ones, since for $\sigma_{x}=\sigma_{y}$. the weighting factors show no dependency on ϕ, and for that case, the analytical formulas are optimal.

The lower subfigures depict the parameters of the covariance matrix ${\underline{R}}_{d\phi }$, again as a function of ϕ comparing different methods. Here, the circles represent numerical results obtained from the definitions of variance and covariance by summing over all $N_{s}$ passes with $1\;\leq \;k\;\leq \;N_{s}$, yielding $d_{k}$ and $\phi_{k}$, respectively:

(57) $$\sigma_{d}^{2}=\frac{1}{N_{s}}\sum_{k=1}^{N_{s}}{\left(d_{k}-d\right)}^{2}$$

(58) $$\sigma_{\phi }^{2}=\frac{1}{N_{s}}\sum_{k=1}^{N_{s}}{\left(\phi_{k}-\phi \right)}^{2}$$

(59) $$\sigma_{d\phi }=\frac{1}{N_{s}}\sum_{k=1}^{N_{s}}\left(d_{k}-d\right)\left(\phi_{k}-\phi \right)$$

Since these numerical results serve just as a reference for judging the accuracy of the error models, in the formulas above, the true values for *d* and ϕ have been used. The required line parameters $d_{k}$ and $\phi_{k}$ in (57)–(59) can be estimated with any of the four described methods, since minor differences in $d_{k}$ and $\phi_{k}$ have almost no effect on the resulting variances and the covariance. The blue lines in Figure 5d–f show the results of the analytic error model as described in Section 3, and the black lines represent the outcomes of the algorithm from Krystek and Anton [35], while the red lines corresponds to the model in closed-form according to (56) in Section 4 with *L* and $x_{off}$ taken from (41) and (49), respectively. Interestingly, although the theoretical derivations differ substantially, the results match very well, which especially proves the correctness of the simplified model in closed-form. Since this model explicitly considers the effect of the line length *L* and of the geometric offset $x_{off}$, the behavior of the curves can be clearly understood: The minimum of *L* will occur if ϕ equals the mean value of $\theta_{min}$ and $\theta_{max}$, i.e., at $\phi =55^{\circ }$, and exactly at this angle, the maximum standard deviation $\sigma_{\phi }$ occurs. Further, since *L* linearly depends on ϕ, a quadratic dependence of $\sigma_{\phi }$ on ϕ according to (47) can be observed. With respect to Figure 5e, the minimum of $\sigma_{d}$ also appears at $\phi =55^{\circ }$ corresponding to $x_{off}=0$. At this angle, according to (54), the standard deviation of *d* is given as $\sigma_{d}\approx \sigma_{\rho }/\sqrt{N}=5/\sqrt{40}=0.79$, while the covariance $\sigma_{\rho d}$ calculated according to (55) and with it the correlation coefficient shown in Figure 5f vanish.

When comparing the results, one should be aware that in the simulations of the analytic error models, the exact variances $\sigma_{x_{i}}^{2}$, $\sigma_{y_{i}}^{2}$ and $\sigma_{xy_{i}}$ are used; thus, in practice, the achievable accuracies will be worse. On the other hand, when applying the new error model in closed-form, the variance $\sigma_{\rho }^{2}$ is calculated as the mean value of all $\rho_{i}^{2}$ from the actual set of *N* data points according to (39), and hence, is always available.

**Figure 6:**
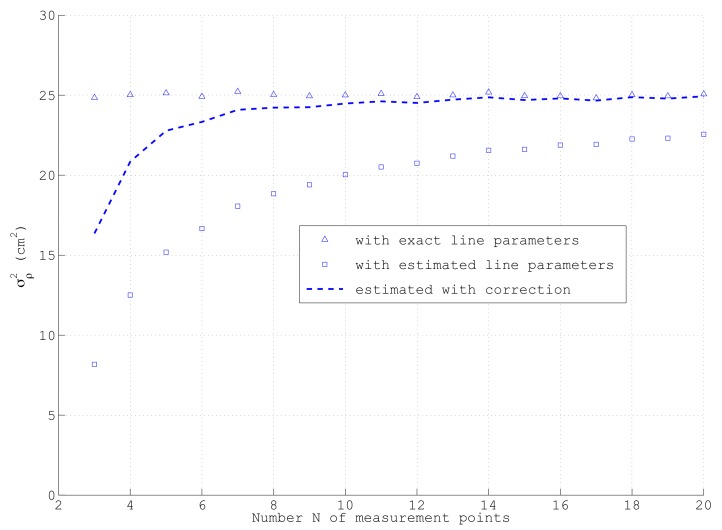
Variance of ρ dependent on the number *N* of measured data points, using the same simulation parameters as indicated in Figure 5a.

Nevertheless, if in this equation, the estimated line parameters ϕ and *d* are used, which are calculated, e.g., according to (3) and (4) using the same measurements as in (39), no unbiased $\sigma_{\rho }^{2}$ can be expected. This is reasoned from the fact that for each set of *N* data points, the mean quadratic distance over all $\rho_{i}^{2}$ is minimized in order to estimate ϕ and *d*. Thus, the numeric value of $\sigma_{\rho }^{2}$ will always be smaller than its correct value calculated with the exact line parameters. This effect can be clearly observed from Figure 6, which shows for the same simulation parameters as depicted in Figure 5a the dependency of $\sigma_{\rho }^{2}$ on the number of points on the line *N*, averaged over $N_{s}$ sets of samples: only in the case of using the exact line parameters in (39), which obviously are only available in a simulation, actually the correct $\sigma_{\rho }^{2}=25\;$ cm${}^{2}$ is obtained as shown by the triangles. If however, in each run, $\sigma_{\rho }^{2}$ is calculated with the estimated ϕ and *d* as indicated by the squares, a clear deviation especially at low *N* occurs. Only asymptotically for large *N* when ϕ converges to its exact value, the correct $\sigma_{\rho }^{2}$ is reached. Fortunately, this error can be compensated quite well by means of multiplying $\sigma_{\rho }^{2}$ with a correction factor $c=\frac{N+1}{N-1}$ as shown by the dashed line in Figure 6. Due to the strongly non-linear relation between ϕ and any $\rho_{i}$, this correction works much better than simply exchanging in (39) the divisor *N* by N−1 as often used in statistics. Since *c* is the inverse of the term neglected in the approximation of $\sigma_{\phi }^{2}$ in (47), the closed-form of the covariance matrix ${\underline{R}}_{d\phi }$ according to (56) yields almost unbiased results also for small *N* if $\sigma_{\rho }^{2}$ is calculated according to (39) with estimated line parameters ϕ and *d*. Although not shown here, the proposed bias compensation works well for a large range of measurement parameters. For a reliable determination of $\sigma_{\rho }^{2}$ from *N* data points of a line segment, *N* should be at least in the order of 10.

**Figure 7:**
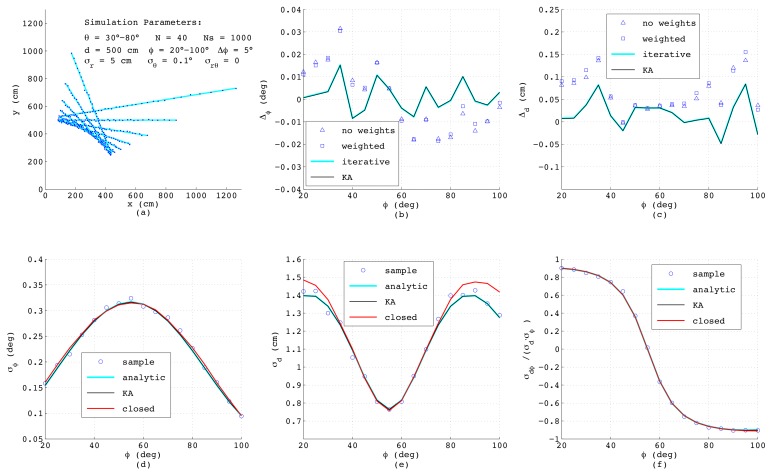
Results from simulated range-bearing scans superimposing low noise in the *r*- and θ-direction.

Figure 7 shows the results when simulating a range-bearing scan with a constant angular offset $\Delta \theta \;=\;\left(\theta_{max}-\theta_{min}\right)/\left(N\;-\;1\right)$ between adjacent measurements. Each measurement is distorted by adding normally-distributed noise with standard deviations $\sigma_{r}\;=\;5\;$cm and $\sigma_{\theta }=0.1^{\circ }$. This is a more challenging situation, since now that the measurements are not equispaced, each data point exhibits individual variances $\sigma_{x,i}$, $\sigma_{y,i}$ dependent on ϕ, and moreover, a covariance $\sigma_{xy,i}$ exists. As can be seen, the errors of the estimated ϕ and *d* as depicted in Figure 7b,c exhibit the same order of magnitude as before; yet, both analytic results differ slightly from each other and are less accurate compared to the numerical solutions. Both numerical methods yield quasi-identical results, since for the chosen small noise amplitudes, the differences between both algorithms have no impact on the resulting accuracy.

Regarding the error models, Figure 7d–f reveal that in spite of unequal distances between the measurement points and varying $\sigma_{\rho ,i}$, the results of the closed-form model match well with the analytic and numeric results. Only $\sigma_{d}$ shows a certain deviation at steep and flat lines with ϕ below $30^{\circ }$ or above $80^{\circ }$. This is related to errors in $x_{off}$, since in this range of ϕ, the points on the lines measured with constant Δθ have clearly varying distances, and thus, (49) yields just an approximation of the effective offset of the straight line.

**Figure 8:**
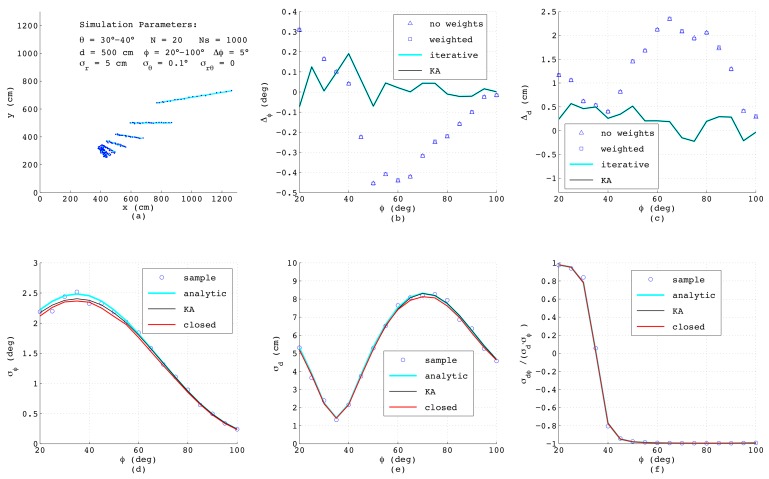
Results from simulated range-bearing scans of short lines superimposing low noise in the *r*- and θ-direction.

The next Figure 8 shows the results with the models applied to short lines measured in the angular range of $30^{\circ }\;\leq \;\theta \;\leq \;40^{\circ }$ with N=20, while all other parameters are identical to those depicted in Figure 7a. As can be seen from Figure 8b,c, now, the analytical algorithms based on (3) and (4) are no longer adequate since these, independent of applying weights or not, yield much higher errors than the numerical approaches. All error models however still provide accurate results. Actually, the closed-form model even yields better accuracy than before, since the distances of the data points on the line between adjacent measurement and also $\sigma_{\rho ,i}$ are more uniform compared to the simulations with long lines.

**Figure 9:**
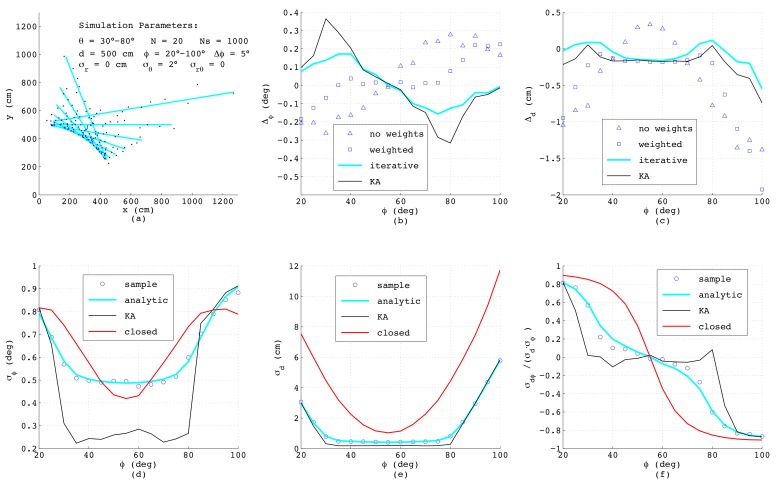
Results from simulated range-bearing scans superimposing high noise only in the θ-direction.

In order to check the limits of the models, Figure 9 depicts the results when applying large angular noise with $\sigma_{\theta }\;=\;2^{\circ }$. In this extreme case, also the numerical algorithms show systematic errors dependent on ϕ since the noise of $\rho_{i}$ can no longer be assumed to be normally distributed. However, according to Figure 8b,c the iterative method as presented in Section 2 shows clear benefits in comparison to the KA algorithm proposed in [35], caused by the more accurate modeling of $\sigma_{\rho_{i}}$.

With respect to the outcome of the noise models in Figure 9d–f, now, only the analytic algorithm as presented in Section 3 still yields reliable results, while the KA-method based on matrix inversion reveals numerical instability. Due to the clear uneven distribution of measurements along the line, also the simplified error model in this case shows clear deviations, although at least the order of magnitude is yet correct.

**Figure 10:**
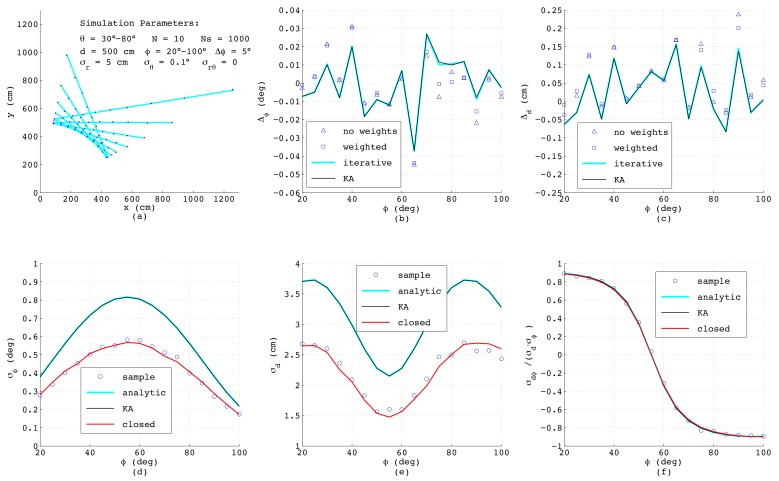
Results from simulated range-bearing scans with a low number of data points and only an approximately known noise level of the sensor.

Finally, Figure 10 shows typical results, if the sensor noise is not exactly known. In this example, the radial standard deviation was assumed to be 10 cm, whereas the exact value, applied when generating the measurements, was only 5 cm. The simulation parameters correspond to those in Figure 7, only the number of data points has been reduced to N=10. According to Figure 10b,c, now, for calculating ϕ and *d*, the numerical methods yield no benefit over the analytical formulas with or without weights. Due to the only approximately known variance, the analytic error model, as well as the KA-method in Figure 10d–f reveal clear deviations from the reference results. Only the model in closed-form is still accurate, since it does not require any a priori information regarding sensor noise. In addition, these results prove the bias-free estimation of $\sigma_{\rho }^{2}$ with (39) also if *N* is low, as depicted in Figure 6.

## 6. Conclusions

In this study, the performance of linear regression is evaluated, assuming both coordinates as random variables. It is shown that, especially with range-bearing sensors, frequently used in mobile robotics, a distinct covariance of the noise in the *x*- and *y*-direction at each measurement point exists. In this case, analytical formulas assuming identical and uncorrelated noise will only provide accurate line parameters ϕ and *d* if the detected line segments are sufficiently long and the noise level stays below a certain limit. If these prerequisites are not fulfilled and if the sensor noise is known, numerical algorithms should be applied, which consider the reliability of each measurement point as a function of ϕ. For this, the performance of prior work can be improved by means of modeling the independence of the single data points exactly and by paying attention also to second order effects of the angular noise.

The main focus of this paper is on the derivation of the covariance matrix ${\underline{R}}_{d\phi }$ of straight lines. This information has a crucial impact on the performance of SLAM with line features, since for both, data association and sensor fusing, ${\underline{R}}_{d\phi }$ must be estimated precisely. For this purpose, the first analytical error models are reviewed, which however need exact knowledge of the measurement noise, although in many applications, this is not available. In addition, these approaches require high computational effort and do not allow one to comprehend the effect of measurement parameters on the resulting accuracy of an estimated straight line. Thus, a new error model in closed form is proposed, depending only on two geometric parameters, as well as on the number of points of a line segment. Besides, a single variance must be known, which is determined easily and reliably from the same measurements as used for line fitting. By means of this model, the covariance matrix can be estimated quickly and exactly. Moreover, it allows one to adapt measurement conditions in order to achieve the maximum accuracy of detected line features.

## Appendix A. Analytic Derivation of Straight Line Parameters With Errors in Both Coordinates

For the derivation of the perpendicular distance *d*, the partial derivative of Equation (2) with respect to *d* is taken and set to zero, which directly gives Equation (4) using (8)–(10). In order to calculate ϕ, first the partial derivation of (2) with respect to ϕ must be calculated and set to zero, yielding:

(A1) $$\frac{1}{N}\sum_{i=1}^{N}s_{i}\left[x_{i}y_{i}\left(\cos^{2}\;\phi \;-\sin^{2}\;\phi \right)+\left(y_{i}^{2}\;-x_{i}^{2}\right)\sin\phi \;\cos\phi \right]+\frac{1}{N}\sum_{i=1}^{N}s_{i}d\left(x_{i}\sin\phi -y_{i}\cos\phi \right)=0$$

Now, the distance *d* can be replaced by (4), and after inserting the definitions of $\bar{x}$, $\bar{y}$, $\sigma_{x}^{2}$, $\sigma_{y}^{2}$ and $\sigma_{xy}$ according to (5)–(9) considering (10), it follows from (A1) after reordering:

(A2) $$\sigma_{xy}\left(\cos^{2}\phi -\sin^{2}\phi \right)+\sin\phi \cos\phi \left(\sigma_{y}^{2}-\sigma_{x}^{2}\right)=0$$

Applying the theorem of Pythagoras and the addition theorems of angles, the terms with the sine and cosine can be rewritten:

(A3) $$\cos^{2}\phi -\sin^{2}\phi =2\cos^{2}\phi -1=\cos2\phi$$

(A4) $$\sin\phi \cos\phi =\frac{1}{2}\sin2\phi$$

Inserting these formulas into (A2) finally yields for ϕ:

(A5) $$\phi =\frac{1}{2}\arctan\left(\frac{-2\sigma_{xy}}{\sigma_{y}^{2}-\sigma_{x}^{2}}\right)$$

Equation (A5) calculates ϕ always in the range −π/4<ϕ<π/4, although according to Figure 1, this is only correct if $\sigma_{y}^{2}>\sigma_{x}^{2}$, while in the case $\sigma_{y}^{2}<\sigma_{x}^{2}$, an angle π/2 must be added to ϕ. Thus, as general solution (3) should be taken also avoiding a special consideration if $\sigma_{y}^{2}$ equals $\sigma_{x}^{2}$.
